# Challenges for consent and community engagement in the conduct of cluster randomized trial among school children in low income settings: experiences from Kenya

**DOI:** 10.1186/1745-6215-14-142

**Published:** 2013-05-16

**Authors:** George Okello, Caroline Jones, Maureen Bonareri, Sarah N Ndegwa, Carlos Mcharo, Juddy Kengo, Kevin Kinyua, Margaret M Dubeck, Katherine E Halliday, Matthew CH Jukes, Sassy Molyneux, Simon J Brooker

**Affiliations:** 1 Health Systems Department, Kenya Medical Research Institute-Wellcome Trust Collaborative Programme, Kilifi, Kenya; 2 Centre for Tropical Medicine, Nuffield Department of Clinical Medicine, University of Oxford, Oxford, UK; 3 Department of Public Health & Primary Care, University of Oxford, Oxford, UK; 4 Malaria Public Health & Epidemiology Group, Kenya Medical Research Institute-Wellcome Trust Collaborative Programme, Nairobi, Kenya; 5 Health and Literacy Intervention Project, Ukunda, Kenya; 6 Ministry of Public Health and Sanitation, District Medical Officer of Health, Msambweni, Kenya; 7 Department of Teacher Education, College of Charleston, Charleston, SC, USA; 8 Faculty of Infectious and Tropical Diseases, London School of Hygiene and Tropical Medicine, London, UK; 9 Graduate School of Education, Harvard University, Cambridge, MA, USA

**Keywords:** Malaria, Cluster-randomized trial, Consent, Community engagement, School-based research, Kenya

## Abstract

**Background:**

There are a number of practical and ethical issues raised in school-based health research, particularly those related to obtaining consent from parents and assent from children. One approach to developing, strengthening, and supporting appropriate consent and assent processes is through community engagement. To date, much of the literature on community engagement in biomedical research has concentrated on community- or hospital-based research, with little documentation, if any, of community engagement in school-based health research. In this paper we discuss our experiences of consent, assent and community engagement in implementing a large school-based cluster randomized trial in rural Kenya.

**Methods:**

Data collected as part of a qualitative study investigating the acceptability of the main trial, focus group discussions with field staff, observations of practice and authors’ experiences are used to: 1) highlight the challenges faced in obtaining assent/consent; and 2) strategies taken to try to both protect participant rights (including to refuse and to withdraw) and ensure the success of the trial.

**Results:**

Early meetings with national, district and local level stakeholders were important in establishing their co-operation and support for the project. Despite this support, both practical and ethical challenges were encountered during consenting and assenting procedures. Our strategy for addressing these challenges focused on improving communication and understanding of the trial, and maintaining dialogue with all the relevant stakeholders throughout the study period.

**Conclusions:**

A range of stakeholders within and beyond schools play a key role in school based health trials. Community entry and information dissemination strategies need careful planning from the outset, and with on-going consultation and feedback mechanisms established in order to identify and address concerns as they arise. We believe our experiences, and the ethical and practical issues and dilemmas encountered, will be of interest for others planning to conduct school-based research in Africa.

**Trial registration:**

National Institute of Health NCT00878007

## Background

There is growing appreciation that good health is important for the education of school-going children. Operational experience in implementing school health and nutrition programmes such as deworming show that they do not serve as a ‘tax on education’, not least because such interventions can be beneficial to educational outcomes and are well received by local communities [1]. Increasingly, more research is being conducted to identify additional interventions that can address the health problems of school-age children [2]. Compared to delivering proven interventions, research conducted in schools on new interventions may cause temporary interruption to instruction while the intervention is being evaluated. There are also a number of practical and ethical issues raised in conducting such research in school settings, including those related to consent/assent and community engagement.

School children aged less than 18 years are considered by many to lack the cognitive maturity and moral development to make individual decisions about research participation [3]. In addition, in many countries they lack the legal competency to enter into binding contracts such as providing individual consent to participate in research [4]. To protect them from potential research exploitation, it is a key ethical requirement that consent be sought from their proxies (parents or legal guardians). In addition, children’s assent must be secured before involving them in research [4].

In developing country settings, challenges in achieving voluntary informed consent among proxies are arguably exacerbated by relatively low exposure to, and familiarity with, science [5] and by a desire to access research-related healthcare in the context of severely resource-constrained public health systems [6]. In these, as in other contexts, proxies may enrol their children into a study out of politeness, or on the basis of a ‘therapeutic misconception’ (where the aim of the study is mistakenly thought to be for the benefit of the individual participant) [7], or because of the actual or perceived benefits of research participation [7,8]. The nature of relationships between researchers and the researched communities, and between genders have also been cited as important determinants of consenting decisions in health research conducted in developing countries [8]. Furthermore, misunderstandings associated with trial procedures such as randomization can lead to distortions and confusions about the process among the community, negative perceptions or raised expectations about the research, and ultimately poorly informed consenting decisions [9-11]. Where decisions are made on behalf of children on the basis of inadequate or incorrect information, a key protection of children may be undermined.

Concerns have been raised that once informed consent is secured from their proxies, children may be included in a study without any further attempts to explain to them the implications of taking part in the study [12], and that researchers and other players in the research process may ignore children’s complaints and signs of discontent [13,14]. Assent in a broader sense refers to the child’s agreement to participate in research after receiving some basic information about the study from the researcher [12]. Increasingly, there are, therefore, requirements for more or less formal assent processes to provide children with basic information about the research and engage them in decision-making regarding research participation [12,15].

One approach to developing, strengthening, and supporting appropriate consent/assent processes is through community engagement. There is no universally accepted definition of community engagement, with descriptions ranging from improving information and transparency, through active consultation, to ensuring greater control or partnership by community members [16]. Community engagement can facilitate the ethical conduct of research, increase understanding of the research process and enhance the acceptance, participation, and retention in research trials [8,17,18]. In addition, it can empower communities to engage on more equal terms with researchers [19], and foster mutual relationships between researchers and communities [17,20]. A number of studies have documented community engagement processes in community/hospital-based research. However, there is still very limited, if any, documentation of community engagement in the context of school-based health research.

The main aim of this article is to share our experiences of implementing a large school-based cluster randomized trial in rural Kenya. The approach taken to community engagement and consent/assent, the main communication issues faced at each stage, and how these were handled, are shared. We conclude with a discussion on some of the ethically relevant dilemmas raised, and with a recommendation for more formal research on consent and engagement processes in these contexts.

## Methods

### The health and literacy intervention trial

#### Trial design

**Figure 1:**
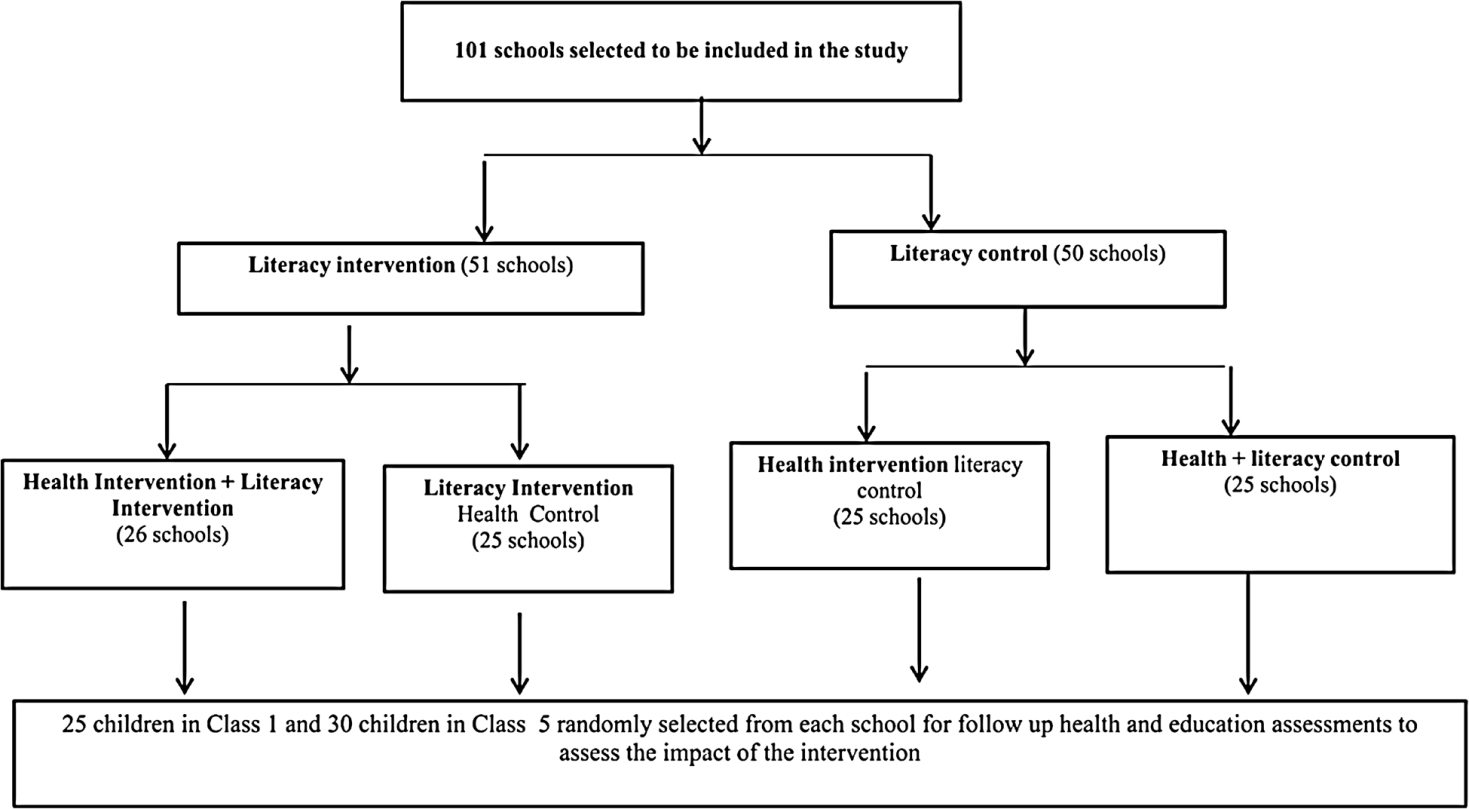
Randomization and participant selection.

The trial design and the complex health and literacy intervention discussed in this paper have been detailed elsewhere (ClinicalTrial.gov: NCT00878007) [21]. In summary, a four-arm cluster randomized trial of the impact of intermittent screening and treatment for malaria and enhanced literacy instruction on health and educational outcomes, was undertaken between January 2010 and March 2012 among pupils initially in class 1 and class 5 in 101 public primary schools. The schools were in the rural districts of Kwale and Msambweni, which are among the poorest in Kenya, and which consistently perform poorly in the national school examinations [21]. The mean age of children participating in this study at baseline assessments was 10.3 years [22]. Schools were randomized to one of four groups: (1) receiving the malaria intervention alone; (2) the literacy intervention alone; (3) both interventions; or (4) control group where neither intervention was implemented (Figure 1) [21]. The malaria intervention involved a mobile team of government health workers (nurses and laboratory technicians) visiting schools to test children for malaria using rapid diagnostic tests (RDTs) and treating all children found to be RDT positive (whether symptomatic or asymptomatic) with artemetherlumefantrine (AL) as per the national guidelines [21,22]. The literacy intervention involved training workshops and support for government primary school teachers to promote explicit and systematic literacy instruction in classrooms. Details of intervention components in each study arm have been summarized in Table 1.

**Table 1:** Intervention components by study arm

**Intervention arm**	**Activities**
1. Malaria intervention	• Testing school children for malaria using rapid diagnostic tests
	• Children testing RDT positive (both symptomatic and asymptomatic) treated using artemetherlumefantrine
	• Height, weight, and hemoglobin concentration also measured at baseline, mid-term, and end-term assessments
	• Educational assessments
2. Literacy intervention	• Teacher training program on improved methods of instruction
	• Instructional materials support
	• Text message support
	• Height, weight, and hemoglobin concentration also measured at baseline, mid-term, and end-term assessments
	• Educational assessments
3. Malaria + literacy intervention	A combination of literacy intervention and malaria intervention activities
4. Control schools	• No literacy or malaria interventions
	• Educational assessments
	• Height, weight, and hemoglobin concentration also measured at baseline, mid-term, and end-term assessments

#### The evaluation team

The trial was implemented by the Kenya Medical Research Institute (KEMRI), a parastatalorganization responsible for carrying out health research in Kenya, in collaboration with investigators from the London School of Hygiene and Tropical Medicine and Harvard Graduate School of Education. The trial team comprised 15 newly employed community mobilizers from the study area, three community liaisons officers, and 38 educational assessors, all with at least secondary school level education. In addition, there were 12 nurses and eight laboratory technologists who were seconded to the project by the district health management team (DHMT) and worked on a rotational basis. The trial team were supervised by two senior study coordinators working directly under the supervision of the principal investigators, and in close collaboration with the DHMT and the district education offices (DEO).

#### The qualitative study

Alongside the health and literacy intervention trial, a qualitative investigation of the acceptability and factors likely to affect the uptake and sustainability of intermittent screening and treatment approach was undertaken. The qualitative study involved 17 in-depth interviews with members of the DHMT and the DEO, and 22 focus group discussions (FGDs) with key stakeholders including parents, teachers, and community health workers [23]. Three additional FGDs were conducted with the field staff (health workers, educational assessors, and community mobilizers) directly involved in trial implementation to gather their views and experiences of the consenting and intervention processes. In this paper, we draw on the data collected during the qualitative study as well as on the authors’ experiences and observations of practice documented over the course of implementing the trial and undertaking community engagement.

#### Ethical approval

Ethical approval for the trial, including associated qualitative work, was provided by the KEMRI and National Ethics Review Committee (SSC No. 1543), the London School of Hygiene and Tropical Medicine Ethics Committee (5503), and the Harvard University Committee on the use of Human Subjects in Research (F17578-101). At an institutional level within Kenya, there are requirements for all studies to carefully consider community engagement as well as consent for all biomedical studies, with guidelines aimed at ensuring appropriate community entry, consultation with community representatives where necessary, and consideration of community sensitization needs and if and how findings will be fed back to participating communities.

## Results

### Process, experiences, and challenges

This section has been divided into three main themes, each of which could be considered a stage in a wider community engagement and consenting process: (1) community entry and randomization; (2) sensitizing and consenting parents and teachers; and (3) informing and assenting children. In presenting our experiences, particular emphasis is placed on the challenges we faced in the conduct of this research and the community engagement activities aimed at overcoming them.

### Community entry and randomization

To conduct the research in Kenyan schools, and to facilitate the uptake of findings, the trial staff engaged with four groups of health and education sector stakeholders at national, provincial, district, and local level. Details of the groups involved, the purpose of engagement, and the challenges faced in engagement are presented in Table 2.

**Table 2:** Stakeholder engagement, goals for engagement, and experiences

**Stakeholders**	**Goals for engagement**	**Challenges**
1. National level stakeholders (Ministry of Health and Ministry of Education)	• Sensitize them about the study	• Unavailability due to busy schedules
	• Seek their opinions about the study design	• Inadequate time to have in-depth discussions about the study
2. Provincial level stakeholders (Ministry of Health and Ministry of Education)	• To identify any study related concerns	
	• Get their endorsement to proceed with the study	
3. District level stakeholders (District education office, district health management team)	• To sensitize them about the study	• Equally busy and at times unavailable for meetings
	• To seek their support to conduct the study in the district	• Because of more involvement in the study, it may be difficult to balance personal interests and expectations with study objectives.
	• To obtain feedback on study design and recruitment strategies	
	• To identify contextual factors that may affect implementation	
	• To map out local stakeholders and assist in mobilization	
4. Local level stakeholders (Chiefs, community health workers, teachers, area education officers, parents’ representatives)	• To sensitize them about the study	• Difficulties in understanding technical study procedures
	• Obtain their support for the study	• Views and perceptions of the study can negatively influence participation
	• Obtain permission to hold meetings in schools and community	• Potential for ‘role slippage’ and risk of ‘coercing’ people to participate in the study
	• Assist in mobilization and information dissemination	• Personal needs might override reasons for engagement which could be linked to incentives
		• Can sabotage the project if not well informed/consulted

At the national level, meetings were held with the National Malaria Control Programme and the Department of Disease Control of the Ministry of Public Health and Sanitation (MoPHS) and with the Director of Basic Education, Director of Quality Assurance and Standards, and School Health and Nutrition unit of the Ministry of Education. Identifying suitable times and organizing the meetings proved challenging. However, once arranged, meetings led to national-level stakeholders from both health and education sectors visiting the study sites to meet with their local counterparts and review project activities. This national-level support, supplemented by formal letters of introduction and support, was very helpful in encouraging district-level involvement.

Meetings at the district level were held with members of the DHMT and the DEO. The key concern that emerged during these meetings was around school selection. Initially, educational officials and district health managers were keen to include the most ‘needy’ schools either because they performed poorly in national examinations, or were more affected by malaria.

To explain and conduct randomization, schools were selected in a public ceremony attended by members of the DHMT and DEO, head teachers, area education officers, and parents’ representatives. The first ceremony selected clusters of schools to be randomly assigned to literacy intervention and control groups while the second meetings randomly assigned schools to malaria intervention and control groups [23]. The ceremony included explanation of the need for stratification and randomization; an explanation assisted through comparison with the pool selection for the 2010 Football World Cup that occurred at the same time. Participants were invited to pick the sealed envelopes containing school names and to place them in education intervention box ‘A’ or ‘B’. At the end, the envelopes were opened by head teachers to see which schools were in which group. As the consenting process was not fully complete at the time of randomisation, the groups were simply known as ‘1’, ‘2’, ‘A’, ‘B’ to the trial team and all stakeholders. There is no word for ‘randomization’ in Swahili (the local language) and it can be a complex concept for individuals to fully comprehend. The participation of district and local stakeholders in the randomization process ensured that they were fully conversant with the process. All aspects of the process were explained to them and their initial concerns and questions about school selection were addressed.

Early meetings with the local level stakeholders directly in charge of the children (local education officials, parents’ representatives, and head teachers) were important in establishing their cooperation and support, and in developing effective lines of communication. This was essential given their gate-keeping roles in the consenting and intervention procedures. Through these meetings, we were also able to identify context specific factors to consider while implementing the trial and additional local level stakeholders (area chiefs, religious leaders, and civic leaders) to engage in the project.

### Sensitization and consent of parents and teachers

#### Sensitization and consent for parents

Before the study and prior to the randomization process, initial meetings were held in schools with the parents (used broadly to refer to guardians as well) of potential participants to describe the purpose of the study, the procedures to be followed, the risks and benefits of participation, and as a first opportunity to obtain informed consent. These meetings were presided over by a team of trained field workers recruited from the local community. Interested parents were invited to provide consent at the end of the meeting. Follow-up school visits targeting parents who attended the initial meeting but requested to be given more time to consult their spouses and other relatives, as well as those who did not attend the first school meeting, were organized 1 to 2 weeks later.

#### Challenges and issues arising from sensitization and consent meetings

In a number of schools, attendance at these sensitization meetings was poor, both at initial and follow-up meetings, necessitating further household visits to obtain adequate and representative numbers of consenting participants in all schools. In rare cases, mothers who provided consent in the first school meeting came to the follow-up meeting to withdraw their consent after their decisions had been overturned by their husbands. This experience illustrates the necessity to consider the dynamics of decision-making, particularly in relation to gender and family relationships, when it comes to recruitment for research participation.

Conducting household visits was equally challenging. In many cases, parents were away during the day, leading to field workers having to make repeated visits. In some instances, study activities (health and educational assessments) had to be rescheduled in schools with very low consent rates, further affecting study timelines and budgets. Nonetheless, household visits were critical to gaining consent from adequate numbers of parents. School meetings and household experiences are summarized in Table 3.

**Table 3:** Participation: obtaining informed consent from parents/guardians

**Activities**	**Advantages**	**Specific challenges**	**Response**
School meetings	• Easy to organize	• Low attendance	• Follow-up school meetings organized
	• Resource and time efficient	• Consenting decisions may be influenced by other parents	• Meetings held outside classrooms to minimize disruption
	• Follow-up consent meetings allowed time for consultations	• Disruption to school schedules	• Insistence on parental consent
		• Danger of proxy consent from relatives	• Household visits
Household visits	• Allows for more in-depth and one-on-one discussions of the study	• Difficulties in tracking households	• Use of village elders/community health workers to track parents
	• Eliminates pressure and influence associated with school meetings	• Unavailability of parents at home	• Field workers sent to households early in the morning or late in the evening
	• Can allow joint decision-making to participate in research	• Time consuming	
		• Resource intensive	

Key issues that emerged during the parental informed consent process included: questions about the rationale for testing and treating apparently healthy children; concerns about blood volumes and the possibility of covert HIV testing; concerns about the safety of AL; the rationale for obtaining individual signed consent for a study that had been approved by the government; and the benefits of participating in the study. These issues have been discussed in details elsewhere [23].

Issues relating to randomization, including the criteria used to select schools and children to participate, and concerns about why some schools or children were not receiving either of the two interventions, also came up during consent meetings and persisted throughout the implementation of the intervention in schools.

*Some people (other parents) were also asking why you came and only enrolled class 1 and 5…Is it that the other children in school don’t have malaria? … Even us parents we ask why not all schools were taken? Is it that they don’t have malaria? Why only two classes? This is what makes us get confused…. We don’t understand it…* (FGD, parents)

That not all children whose parents had provided consent received an intervention (either literacy or malaria or both) was both difficult to understand or accept.

*You see there were parents who consented but their names did not appear in our lists. So some parents would come and complain. They would ask why they were asked to provide consent and even followed up during household visits (to provide consent) if their children were not going to take part in the study…* (FGD, education assessors)

To address some of the above concerns, a more proactive approach to community sensitization was adopted. For instance, to address parents’ fears about the possibility of covert HIV testing and the safety of study drugs, field workers carried samples of malaria RDTs and AL and showed them to participants. Field workers emphasized that AL was safe and had been approved for routine use in Kenya. Parents were also encouraged to come to schools to witness the screening and treatment process to address their fears about blood volumes. To address the concerns raised by the randomization process, fieldworkers were encouraged to discuss the scientific rationale and benefits of the randomization process and to address rather than avoid the concerns of parents and teachers as they arose. In addition, a community liaison group comprising of three experienced field workers was formed to act as the link between the research team and the community. They were responsible for identifying contentious issues about the research process in the community and responding to them. Similarly, the liaisons group accompanied health teams to schools and responded to any concerns that emerged during the exercise. Through the community liaison group, we were able to maintain dialogue with parents/guardians and the community throughout the study period.

#### Sensitization and consent for teachers

The process for sensitizing the teachers involved in the literacy intervention and acquiring their approval was conducted on two tracts and at several time points. The initial permission to have teachers deliver the literacy intervention in schools was granted by the Ministry of Education. Senior education officials perceived that the individual consent of teachers to deliver the intervention was not required as it was seen to be part of routine classroom instruction. As such, we did not seek individual consent for teachers to participate in the literacy intervention. However, all teachers in the participating schools were encouraged to attend the school sensitization meetings in the months preceding the randomization, to learn about the study. Furthermore, the first activity of the teacher training for the delivery of the literacy intervention was a detailed explanation of the intervention including how it was developed with the input of local teachers. This explanation was accompanied by a letter summarizing the intervention and stating that their participation in the delivery of the intervention was voluntary. No teachers opted out of the training. Individual consent was also obtained from them teachers whenever they were the subject of assessment exercises or classroom observations.

#### Issues around sensitization and consent for teachers

The support of teachers was critical both during the informed consent and sensitization meetings and the implementation of the intervention in schools. In some schools, however, field workers reported the existence of tension in their relationships with teachers, particularly those who were not involved in the delivery of the literacy intervention. These tensions appeared to emanate from these teachers feeling excluded from critical aspects of the project such as the information dissemination campaigns described above, which focused on parents, head teachers, intervention teachers, and other stakeholders. These teachers felt that parents entrusted them with the care of their children while in school and expected them to have detailed information about the study. Fieldworkers perceived that without this information, when they were questioned about the study by parents, they were either unable to respond to the questions or may have given inaccurate answers, potentially contributing to withdrawals.

*You see the problem of withdrawals in most cases was caused by teachers. The teachers were supposed to act as the link between us and the parents. So in our absence, he was supposed to give the right information about the study. So parents would ask them questions about the study and some of them would not give the right answers. For instance, some claimed that they did not understand what we were doing. Some told parents that they were just being used. Teachers wanted to be involved more. I remember in one school, a teacher refused to help us mobilize children because he requested us to give him a bottle of water which we were unable to give him because we only had two bottles*… (FGD, community mobilizers)

Communication about the scheduled visits to schools was mainly channeled through the head teacher, who was expected to share this information with other teachers. Sometimes head teachers failed to inform teachers, which led, in several cases to study activities having to be postponed due to teachers refusing to participate in inadequately communicated or timed activities.

*I think at times there was a breakdown in communication. The head teacher would be called but at times, he would not be in school and therefore forgets to pass the same information to other teachers. So teachers in school would not be aware of what is happening…* (FGD, education assessors)

To address such concerns a general meeting targeting deputy head teachers and health teachers from all participating schools was organized. Participants were briefed about the study and requested to share the information with their pupils as well as other colleagues. Following the meeting, in addition to the head teachers, deputy headteachers and health teachers were incorporated into any subsequent communication with schools, receiving text messages to make them aware of the visits. Letters summarizing the previous term’s activities and those scheduled for the new term were also sent out to all schools at the beginning of each term to be pinned on school notice boards for all teachers to see.

The issue of reward and compensation also caused tension in the relationship between field staff and teachers. While the Ministry of Education officials regarded the literacy intervention as part of normal classroom instructions, some intervention teachers cited the additional preparation required for these lessons and requested that they be compensated for their time. For the health assessments, teachers were often required to help the field staff retrieve children from their classrooms and take them to the location in the school where the malaria intervention was being administered. At times, a teacher’s presence throughout the health assessments was also necessary but they were not compensated for their time. Some of them complained about the inability of the project to recognize and reward their efforts; they became unwilling to support field staff during subsequent school visits.

*We understand that KEMRI is an organization that has a lot of money. This is what we believe because we see you travel in very expensive vehicles. So we think it is wise that you give us a token when we do your job because in one way or another, we are professionals. We are teachers. We are not nurses…* (FGD, teachers*)*

Field workers also reported that teachers in the control schools were less supportive of the study after learning that they would not be receiving any intervention but would be expected to take part in evaluation activities (education and health assessments).

*In some schools, teachers told us that we should not disturb them if their schools were control schools because there are no benefits that they were going to receive from taking part in the study.* (FGD, education assessors)

### Informing and assenting children

#### Obtaining assent from children

Verbal assent was obtained from children at the time of baseline educational and health assessments and during subsequent study visits. For educational assessments, assent was obtained by an educational assessor reading out to children an information sheet detailing the various tasks and provision of an assurance that participation was voluntary. Similarly, health workers on the day of intervention and assessments activities explained to children the purpose of the study, and the biomedical procedures involved.

#### Issues in obtaining assent from children

Many children assented verbally after their parents’ consent, but a small minority dissented verbally or non-verbally. Such children sometimes refused to go to the screening rooms, cried or refused the finger prick or anthropometric measurements, or to take medication after testing positive for malaria [23]. In a few schools, some children ran away from their classrooms when study teams arrived. This was mainly common with children in lower classes early on in the study. In older classes, one child refusing to take part in the health assessment would sometimes lead to others following. Older children who dissented from the health assessments often reported that their parents had instructed them not to take part in the study, although these parents had not officially withdrawn.

*You see the children in the lower classes mainly refused because of the fear of being pricked… (For) the ones in upper classes, it was because of adolescence. Some of them felt that they were old enough to make their own decisions…the older ones would refuse even if their parent had provided consent…* (FGD, community mobilizers)

This dissent was not always accepted by teachers or parents, who tried to persuade children to participate against their will, an approach that was strongly discouraged by the field staff. In some cases, parents returned children who had run away from school and insisted they be tested in their presence. In our settings where respect for those in authority is emphasized, field teams faced a dilemma on whether or not to support the dissenting children against their parents or teachers. Supporting dissenting activities based on unnecessary fear and concern could have undermined the success of the trial as well as allowed concerns to persist. On the other hand, ignoring children’s dissent could have undermined their ability to make autonomous decisions to participate in the study or not. For this reason, the team increasingly focused on improving communication, understanding, and familiarity among the children themselves. One such initiative involved teachers and health workers taking finger pricks in front of children to allay their fears about the process. The trial team also constantly reminded parents and teachers that children had a right to refuse to participate in the study. They did not test those children who openly expressed signs of dissent. Dissent reduced as the study progressed perhaps as a result of repeated interactions with trial teams and familiarity with the study procedures.

## Discussion

Our experiences in implementing this large, complex cluster randomized trial involving both a biomedical and a non-biomedical intervention have illustrated the complexity of consent and community engagement processes in school based research. While we did not conduct a formal evaluation of the consent and community engagement process, we believe that our approach in dealing with the challenges that we encountered while undertaking this trial played a major role in ensuring its successful implementation. We were able to implement the trial as planned, with no major issues arising and with tremendous support from local communities as well as from district and national officials. Through constant communication and feedback with key stakeholders we were able to promptly identify and respond to community concerns about the study. Most school-based intervention trials will not be as diverse and complex as ours, but the issues we faced are likely to be relevant to less complex school-based health and education intervention trials.

One such issue relates to the dilemmas of obtaining assent from children in school-based research. While we did not evaluate this directly, some of the dissenting behaviors exhibited by children in this study may have resulted from their (mis)perceptions about the trial procedures and the interventions, and lack of understanding of the purpose of the activities, in part because sensitization initially focused on parents/guardians and other stakeholders as opposed to the children themselves. In the absence of proper information about the study, it is possible that the child’s refusal was influenced by inaccurate rumors [23] and, therefore, unnecessarily heightened fears and anxieties about the trial (especially health interventions). In addition, unequal power relations between children and other players in the research process may have led to less dissent. Assent in this context therefore may have resulted from ‘hidden social pressure’ on children to conform, to avoid reprisal from parents/guardians or teachers [24]. This suggests voluntariness among children and freedom in decision-making, both key pillars of consent processes may have been compromised for some. These experiences have demonstrated the need to provide children with simplified information about the study and actively involve them in wider community engagement activities, alongside parents to improve their understanding of the research process and facilitate assent/dissent decisions.

In terms of facilitating free choice among parents, there was some potential for undermining of voluntariness in both school meetings and home visits. While school meetings were perhaps the most resource and time efficient approach to obtaining consent, the ‘communal nature’ of these meetings did not allow for one-on-one discussions with fieldworkers to strengthen understanding before decision-making. One-on-one discussions may have led to greater understanding, and may also have reduced influence by other parents at these meetings. On the other hand, household visits which potentially overcame some of these challenges, are extremely time-consuming, and may have left some participants feeling pressured by trial staff to allow their child to participate. There are challenges with both approaches and our experiences illustrate the need, when planning the consenting process, to consider strategies to minimize the pressure parents may feel. Such strategies might include providing opportunities for one-on-one discussions in school meetings, limiting the number of visits to any one household, and taking care to minimize persuasion in those discussions.

In terms of understanding, parents and teachers were not always clear about the nature and purpose of the intervention and evaluation activities. Given both the different nature of the two interventions and the complexity of participant selection and the consenting process, this is perhaps not surprising. Lack of clarity about the trial design, particularly about the cluster randomization process and the selection of schools and individual children, may have contributed to concerns about the trial, and to disappointments among parents and teachers when their children/schools were allocated to control groups [25]. Featherstone and colleagues note that where understanding of randomization as a concept is difficult, distortions may occur and participants may form alternative accounts of the process [26].

Our experience in implementing this trial has also highlighted the ethical issues posed by cluster randomized trials more specifically [27]. One such issue relates to the definition of human research subjects in cluster randomized trials especially when the unit of randomization, experimentation and observation are different [28]. In our case, for instance, teachers were trained to deliver the intervention in their classrooms but data to evaluate the outcome of the literacy intervention were collected from their pupils. McRae and colleagues argue that a human research subject in cluster randomized trial needs to be directly intervened upon by the investigator, interact with the investigators, be deliberately intervened upon by the investigator via manipulation of their environment, or provide identifiable private information [28]. To some extent, training of teachers and provision of additional learning resources constituted a direct manipulation of teaching methodology and learning environments in classrooms. Based on the above definition, all literacy intervention teachers therefore qualified as human research subjects and we should have obtained individual consent from them to participate in the intervention as was the case whenever they were the subject of assessment exercises or classroom observations. However, obtaining individual consent from teachers would have also had an impact on the implementation of the study especially if a teacher in an intervention school refused. Randomization for the literacy intervention was done at the cluster level. A cluster consists of between three to seven schools in one geographical area. For the successful implementation of the study, all class teachers in the intervention schools within the cluster were expected to participate in the delivery of the intervention in their schools/classrooms. Dissent would have made it practically impossible to implement the intervention in that school.

The other ethical issue encountered in this trial relates to the role and authority of gatekeepers in cluster randomized trials [29]. Education officials at the national, district, and local levels provided permission for the study to be implemented in schools. They also provided permission for teachers to be trained to deliver the intervention in schools. They perceived that the intervention was part of teachers’ normal duties and individual consent from teachers to participate in its delivery was not necessary. Gallo and colleagues note that gatekeepers can play a major role in controlling access to organizations such as schools by providing permission for researchers to conduct cluster randomized trials using their facilities, resources, and personnel. However, they emphasize that gatekeepers do not have the authority to provide proxy consent for individual cluster members to participate in a study [29]. Given that permission to use teachers to deliver the literacy intervention had been provided by their superiors, teachers had very little room to refuse. Dissent would potentially have been equated with insubordination. This suggests that in the hierarchical workplace, voluntariness in consent can be compromised. In our case, while no teacher declined to attend the literacy intervention training, there were ‘silent refusals’ from some teachers who after attending the teacher training, failed to implement the intervention in their classrooms as per protocol. According to Kamuya and colleagues, ‘silent refusals’ is a situation where participants participate inconsistently, or participate in some study procedures and not others without openly refusing or withdrawing from the study [30]. In this study, ‘silent refusals’ may have been used by these teachers to mask genuine refusals and thereby safeguard important relationships with their superiors and intervention teams [30]. That all children in the literacy intervention classrooms received the literacy intervention regardless of whether their parents had consented to it or not further illustrates the potential loss of individual autonomy in cluster randomized trials where avoiding exposure to interventions delivered at the cluster level may not be possible [31]. Such ethical issues in cluster randomized trials are complex and there is still no consensus on how best to handle them. A recently concluded project by the Canadian Institute of Health Research has issued some guidelines on the ethical design and conduct of cluster randomized trials [32].

Nonetheless, our experiences in implementing this trial have shown that teachers occupy a central position in school-based health research and their active involvement in all aspects of any school-based intervention is critical to its success. They can play a major role in reviewing various study materials such as information sheets, identity, and raise potential concerns about the study and participate in information dissemination. Allowing teachers to freely make active decisions regarding study participation may both strengthen the ethical conduct of the research and also enable them to prepare for the inevitable disruptions that the research process involves [33]. Concerns about lack of remuneration raised by intervention and non-intervention teachers had the potential to undermine the effective delivery of the literacy intervention and the overall implementation of the study in schools. There were no direct monetary benefits to teachers for implementing various study activities in schools. However, intervention teachers received a small weekly mobile airtime top-up to enable them to respond to weekly text messages. Teachers also received additional learning materials to support classroom instruction. We did not provide any compensation to intervention teachers largely because the intervention was delivered as part of normal classroom instruction. However, as has been noted elsewhere [33], teachers may need to be compensated for their involvement in study activities especially if this occurs outside normal class hours [33]. For future studies, we agree with Ross and colleagues that providing teachers with a range of small but meaningful incentives may be an appropriate way of improving study participation and acceptance [34].

## Conclusions

Our experiences in implementing this complex trial have highlighted the importance of adequate consent and community engagement processes in school-based research. Given the range of key stakeholders within and beyond schools, having a dedicated team of liaison staff can be helpful in obtaining feedback and addressing community concerns about the study. These activities require carefully planning and budgeting with appropriate community entry, sensitization, consultation, and feedback mechanisms established to help ensure the successful implementation of school-based trials. The need for careful planning has to be counterbalanced by recognition that community engagement must be responsive to issues and concerns raised through interactions with key stakeholders, and therefore be dynamic and evolve over time.

## Competing interests

The authors declare that they have no competing interests.

## Authors’ contributions

GO, CJ, and SJB conceived the idea of the qualitative study. GO led the data collection, data analysis and developed the manuscript. KEH, CM, JK, MB, and SNN were responsible for field work supervision and data collection of the main trial and contributed to the final manuscript. MCHJ and SJB were responsible for the design of the main trial. MCHJ and MMD were responsible for the design and implementation of the educational assessments. CJ and SM contributed to the interpretation of the results and writing of the manuscript. SJB was responsible for the overall project management and contributed to the interpretation and writing of the manuscript. All authors read and approved the final manuscript.

## References

[B1] Bundy D Rethinking School Health: A Key component of Education for All 2011 Washington, DC: World Bank

[B2] Brooker S Kolaczinski JH Gitonga CW Noo AM Snow RW The use of schools for malaria surveillance and programme evaluation in Africa Malar J 2009 8 231 10.1186/1475-2875-8-231 19840372 PMC2768743

[B3] Coyne I Research with children and young people: the issue of parental (proxy) consent Children Soc 2010 24 227 237

[B4] Council for International Organizations of Medical Sciences (CIOMS) International Ethical Guidelines for Biomedical Research Involving Human Subjects 2002 Geneva: CIOMS 14983848

[B5] Molyneux S Wassenaar DR Peshu N Marsh K ‘Even if they ask you to stand by a tree all day, you will have to do it (laughter)!’: community voices on the notion and practice of informed consent for biomedical research in developing countries SocSci Med 2005 61 443 454 10.1016/j.socscimed.2004.12.003 15893058

[B6] Molyneux S Peshu N Marsh K Understanding of informed consent in a low-income setting: three case studies from the Kenyan coast Soc Sci Med 2004 59 2547 2559 10.1016/j.socscimed.2004.03.037 15474208

[B7] Kamuya D Marsh V Molyneux S What we learned about voluntariness and consent: incorporating “background situations” and understanding into analyses Am J Bioeth 2011 11 31 33 21806436 10.1080/15265161.2011.583328

[B8] Marsh V Kamuya D Rowab Y Gikonyo C Molyneux S Beginning community engagement at a busy biomedical research programme: experiences from the KEMRI CGMRC-Wellcome Trust Research Programme, Kilifi, Kenya Soc Sci Med 2008 67 721 733 10.1016/j.socscimed.2008.02.007 18375028 PMC2682178

[B9] Kerr C Robinson E Stevens A Braunholtz D Edwards S Lilford R Randomisation in trials: do potential trial participants understand it and find it acceptable? J Med Ethics 2004 30 80 84 10.1136/jme.2002.001123 14872081 PMC1757143

[B10] Donovan JL Random allocation or allocation at random? Patients’ perspectives of participation in a randomised controlled trial Br Med J 1998 317 1177 1180 10.1136/bmj.317.7167.1177 9794849 PMC28698

[B11] Robinson E Kerr CEP Stevens AJ Lilford RJ Braunholtz DA Edwards SJ Beck SR Rowley MG Lay public’s understanding of equipoise and randomisation in randomised controlled trials Health Technol Assess 2005 9 1 192 15763039 10.3310/hta9080

[B12] O’Lonergan T Zodrow JJ Pediatric assent: subject protection issues among adolescent females enrolled in research J Law Med Ethics 2006 34 451 459 10.1111/j.1748-720X.2006.00051.x 16789967

[B13] Lott JP Module three: vulnerable/special participant populations Dev World Bioeth 2005 5 30 54 10.1111/j.1471-8847.2005.00101.x 15748177

[B14] Tessa J Hope T Savulescu J Stein A Pollard AJ Children’s consent and paediatric research: is it appropriate for healthy children to be the decision-makers in clinical research? Arch Dis Child 2009 93 379 383 10.1136/adc.2007.118299 18089635

[B15] Broome ME Stieglitz KA The consent process and children Res Nurs Health 1992 15 147 152 10.1002/nur.4770150209 1565807

[B16] Tindana P Singh JA Tracy SC Upshur REG Daar AS Singer PA Frohlich J Lavery JV Grand challenges in global health: community engagement in research in developing countries PLoS Med 2007 4 e273 10.1371/journal.pmed.0040273 17850178 PMC1989740

[B17] Lavery J Tindana PO Scott TW Harrington LC Ramsey JM Ytuarte-Nunez C James AA Towards a framework for community engagement in global health research Trends Parasitol 2010 26 279 283 10.1016/j.pt.2010.02.009 20299285

[B18] Tindana P Rozmovits L Boulanger RF Bandewar SVS Aborigo RA Hodgson AVO Kolopack P Lavery JV Aligning community engagement with traditional authority structures in global health research: a case study from northern Ghana Am J Public Health 2011 101 1857 1867 10.2105/AJPH.2011.300203 21852635 PMC3222376

[B19] Lang T Gould J Seidlein Von L Lusingu JP Mshamu S Ismael S Liheluka E Kamuya D Mwachiro D Olotu A Njuguna P Bejon P Marsh V Molyneux C Approaching the community about screening children for a multicentre malaria vaccine trial Int Health 2012 4 47 54 10.1016/j.inhe.2011.10.003 24030880

[B20] Nyika A Chilengi R Ishengoma D Mtenga S Thera MA Sissoko MS Lusingu J Tiono AB Doumbo O Sirima SB Lemnge M Kilama WL Engaging diverse communities participating in clinical trials: case examples from across Africa Malar J 2010 9 86 10.1186/1475-2875-9-86 20346126 PMC2907873

[B21] Brooker S Okello G Njagi K Dubeck MM Halliday EK Inyega H Jukes MCH Improving educational achievement and anaemia of school children: design of a cluster randomised trial of school-based malaria prevention and enhanced literacy instruction in Kenya Trials 2010 11 93 10.1186/1745-6215-11-93 20929566 PMC2959045

[B22] Halliday E Karanja P Turner EL Okello G Njagi K Dubeck MM Allen E Jukes MCH Brooker S *Plasmodium falciparum*, anaemia and cognitive and educational performance among school children in an area of moderate malaria transmission: baseline results of a cluster randomised controlled trial on the coast of Kenya Trop Med Int Health 2012 17 532 549 10.1111/j.1365-3156.2012.02971.x 22950512 PMC3506732

[B23] Okello G Ndegwa SN Halliday KE Hanson K Brooker SJ Jones C Local perceptions of intermittent screening and treatment for malaria in school children on the south coast of Kenya Malar J 2012 11 185 10.1186/1475-2875-11-185 22681850 PMC3422207

[B24] David M Children and school-based research: ‘informed consent’ or ‘educated consent’? British Educational Research Journal 2001 27 347 365 10.1080/01411920120048340

[B25] Snowdon C Garcia J Elbourne D Making sense of randomization; responses of parents of critically ill babies to random allocation of treatment in a clinical trial Soc Sci Med 1997 45 1337 1355 10.1016/S0277-9536(97)00063-4 9351153

[B26] Featherstone K Donovan JL “Why don’t they just tell me straight, why allocate it?” The struggle to make sense of participating in a randomised controlled trial Soc Sci Med 2002 55 709 719 10.1016/S0277-9536(01)00197-6 12190265

[B27] Weijer C Grimshaw JM Taljaard M Binik A Boruch R Brehaut JC Donner A Eccles MP Gallo A McRae AD Saginur R Zwarenstein M Ethical issues posed by cluster randomized trials in health research Trials 2011 12 100 10.1186/1745-6215-12-100 21507237 PMC3107798

[B28] McRae J Weijer C Binik A White A Grimshaw JM Boruch R Brehaut JC Donner A Eccles MP Saginur R Zwarenstein M Taljaard M Who is the research subject in cluster randomized trials in health research? Trials 2011 12 183 10.1186/1745-6215-12-183 21791064 PMC3162904

[B29] Gallo A Weijer C White A Grimshaw JM Boruch R Brehaut JC Donner A Eccles MP McRae AD Saginur R Zwarenstein M Taljaard M What is the role and authority of gatekeepers in cluster randomized trials in health research? Trials 2012 13 116 10.1186/1745-6215-13-116 22834691 PMC3443001

[B30] Kamuya D Theobald S Munyoki P Koech D Geissler W Molyneux S Evolving friendships and shifting ethical dilemmas: fieldworkers’ experiences in a short term community based study in Kenya Dev World Bioeth 2013 13 1 9 10.1111/dewb.12009 23433316 PMC3662996

[B31] McRae A Weijer C Binik A Grimshaw JM Boruch R Brehaut JC Donner A Eccles MP Saginur R White A Taljaard M When is informed consent required in cluster randomized trials in health research? Trials 2011 12 202 10.1186/1745-6215-12-202 21906277 PMC3184061

[B32] Weijer C Grimshaw JM Eccles MP McRae AD White A Brehaut JC Taljaard M The Ottawa statement on the ethical design and conduct of cluster randomized trials PLoS Med 2012 9 e1001346 10.1371/journal.pmed.1001346 23185138 PMC3502500

[B33] Alibali M Nathan MJ Conducting research in schools: a practical guide J Cogn Dev 2010 11 397 407 10.1080/15248372.2010.516417

[B34] Ross JG Sundberg EC Flint KH Informed consent in school health research: why, how, and making it easy J Sch Health 1999 69 171 176 10.1111/j.1746-1561.1999.tb06378.x 10363220

